# A meta-science for a global bioethics and biomedicine

**DOI:** 10.1186/s13010-017-0051-y

**Published:** 2017-11-07

**Authors:** David S. Basser

**Affiliations:** Philosophy, School of Humanities, University of Tasmania, Hobart, TAS Australia

**Keywords:** Communitarian cosmopolitan, Digital biomedicine, Discernment continuum, Global bioethics, Global biomedicine, Global systemic shift, Integrative biomedicine, Meta-science, Neuroethics, Telos of humankind

## Abstract

**Background:**

As suggested by Shook and Giordano, understanding and therefore addressing the urgent international governance issues around globalizing bio-medical/technology research and applications is limited by the perception of the underlying science.

**Methods:**

A philosophical methodology is used, based on novel and classical philosophical reflection upon existent literature, clinical wisdoms and narrative theory to discover a meta-science and telos of humankind for the development of a relevant and defendable global biomedical bioethics.

**Results:**

In this article, through pondering an integrative systems approach, I propose a biomedical model that may provide Western biomedicine with leadership and interesting insight into the unity beyond the artificial boundaries of its traditional divisions and the limit between physiological and pathological situations (health and disease). A unified biomedicine, as scientific foundation, might then provide the basis for dissolution of similar reflected boundaries within bioethics. A principled and communitarian cosmopolitan bioethics may then be synonymous with a recently proposed principled and communitarian cosmopolitan neuroethics based on a novel objective meta-ethics. In an attempt to help facilitate equal and inclusive participation in inter-, multi-, and transdisciplinary intercultural discourse regarding the aforementioned international governance issues, I offer: (1) a meta-science derived through considering the general behaviour of activity, plasticity and balance in biology and; (2) a novel thought framework to encourage and enhance the ability for self-evaluation, self-criticism, and self-revision aimed at broadening perspective, as well as acknowledging and responding to the strengths and limitations of extant knowledge.

**Conclusions:**

Through classical philosophical reflection, I evolve a theory of medicine to discover a telos of humankind which in turn provides an ‘internal’ moral grounding for a proposed global biomedical bioethics.

## Background

In answer to the calls for a global neuroethics relevant to upgrading international policies and laws dealing with brain research and the uses of novel neurotechnologies, Shook and Giordano, [1] and Lanzilao et al. [2] proposed a principled and cosmopolitan neuroethics based on a novel objective meta-ethics. Inspired by and aligning with Beauchamp and Childress’ model of Principalism [3] they began with the four principles beneficence, non-maleficence, respect for autonomy, and justice. In their model beneficence evolves into empowerment, “so as to advance the capability of people to independently pursue their own well-being with the ultimate purpose to fulfil their lives but not in ways that impugn the freedom – and powers – of others”; non-maleficence evolves into non-obsolescence, “a more proactive duty to sustain individual worth and value within society”; respect for autonomy evolves into self-creativity, “the right of persons to recreate themselves to pursue enrichment in their lives”; justice evolves into citizenship, the ability “to be a free, equal, law-abiding, and participatory citizen” [2]. The science was the starting point and the foundation for their development and, through pondering a unified principled stance, the philosophical path from ‘synapse to society’ lead to their proposal. It was claimed that “neuroethics can find its philosophical foundations in much the same way that its scientific foundations are found in understanding the human brain” and that “the objectivity of the new meta-ethics for neuroethics cannot exceed the degree of scientific objectivity involved” [1]. It was stated that “without doubt neuroethics can and should be seen as a field of ethics, and a sub-field of bioethics” and that “its broad and interdisciplinary applications can foster a systematic interdisciplinarity and an ability to move beyond limitations of western philosophy” [1, 2].

Potter argued a similar need for a global bioethics that is worldwide in scope, unified and comprehensive, encompassing traditional (medical) ethics, ecological concerns and the larger problems of society [4].

I assert that pondering unified principles of biomedicine derived from the Western model of integrative systems biology may: (1) provide the scientific foundation for an internationally relevant (global) neuroethics to become synonymous with, rather than a sub-field of, an internationally relevant (global) bioethics; (2) lead to a meta-science as a means for facilitating an inclusive, pluralist discussion that transcends culture, language, discipline and philosophical boundaries and; (3) lead to a philosophical language framework to facilitate the integration of inter-, multi- and transdisciplinarity beyond any imperialist boundaries including hegemony and absolutism [2]. The resulting unified principles may then address new and future ethical issues arising from emerging biomedical understandings and biotechnologies, including neurotechnologies [5–7].

## Results and discussion

### In search of the scientific view for bioethics: Integrative biomedicine

As indicated in the introduction, according to Shook and Giordano [1], scientific foundation has to play a key role in founding appropriate cosmopolitan ethical approaches inclusive of those applying to novel technologies. However, a legitimate question arises as to what biomedical scientific model, or put simply, what biomedicine view should inform adequate ethical approaches? I assert that an internationally relevant biomedicine must comport with the same four principles prescribed for an internationally relevant neuroethics: “1) it must be sensitive to pluralist views and be liberated from prior hegemonic ideologies; 2) it must fully represent the contemporary reality of the bio-psychosocial human being, as reciprocally engaged in and engaged by human ecology; 3) it must embrace aspects of both individual and collective identity; and 4) it must observe standards of objectivity sufficient for broadly justifying practical [biomedical] positions” [2].

Informed by the conceptualization of dimensions of biomedical understanding as intelligible spheres, where each centre of discernment (beginning) expands to incorporate all others (I detail this conceptual model later in this essay), I begin with the Western biomedical understanding that is psychoneuroimmunology. By addressing the integrated nature of the relationships among behavioral, neural, endocrine, and immune responses that enable an organism to adapt to the environment in which it lives [8] psychoneuroimmunology research has provided biomedicine with leadership and interesting insight into the unity beyond the artificial boundaries of its traditional divisions and the limit between physiological and pathological situations (health and disease) [9]. Effectively, activity in one area is activity in the whole and the expression of that activity in any individual will be determined by the in toto nature of that same individual. In simplified terms, the nervous, endocrine and immune systems are but aspects of a unified integrated whole where, in real conditions, activity in one aspect is activity in all aspects, imbalance in one aspect is imbalance in all aspects, and the principles governing any one aspect govern all.^1^ Accordingly, if we accept the claim that psychoneuroimmunology research offers an integrative foundation, then we defend that this integrative understanding can inform the entire spectrum of bioethics including neuroethics. Supporting and sustaining this is the bio-psychosocial [biomedical] model, originally formulated by Engel [10] and enhanced by Borrell-Carrió, Suchmam & Epstein [11]. These latter authors state that knowledge is a social construct and that categories, such as “mind” or “body” (nervous, immune and endocrine systems) “are useful to the extent that they focus our thinking and actions in helpful ways”…“but when taken too literally they can entrap and limit us by creating boundaries that do not exist.” I propose that the corollary to the dissolution of the socially constructed traditional scientific barriers is the dissolution of the similar reflected boundaries within bioethics. A unified biomedicine as the foundation for a unified bioethics, where any aspect of each will inform the corresponding whole. Here the principles of any and each aspect will also be those of the whole, and a principled and communitarian cosmopolitan neuroethics [1, 2] becomes synonymous with a principled and communitarian cosmopolitan bioethics.

### Meta-science – The intersection point of a globalizing biomedicine

Benedikter et al. [12] stated that “a new (technologic) imperative must acknowledge and comport with a rational understanding of how our biology gives rise to, and is affected by the intersecting artifacts of society and machination (BioSoMa)”. They called for a proactive acknowledgement of BioSoMa that is “conjoined to an understanding of our history, who we are, and the projections of who/what we want to become in the future” in order to address the challenges of the potential future effects of biotechnology. This “necessitates integrating philosophical, anthropological, sociological and theological perspectives with those of (geno-, nano-, neuro- [and broader biomedical]) science to more fully elucidate the basis of our experiences, cultures, beliefs and being, and afford better perspective on the possibilities of the future”. Different fields of inquiry view these questions and issues differently therefore for balanced reflection each and all disciplines should participate as equal members in the discourse. The pursuit of such reflection can be called “ethics”. Ethics can then be defined as “the search for balance and inclusion” and considered a qualitative-quantitative attitude in the field of systemic interaction [12]. These same authors stated that “if the pathway forward is through inquiry, then it is necessary to integrate scientific efforts with trans-disciplinary discourse that aims to (1) shape ethical conduct in research, practice and social domains, and (2) ensure and direct applications of scientific developments toward realising and sustaining the public good. Such tasks … requires open exchange of ideas among groups … from the sciences and humanities”. Open exchange will require an intersection point of understanding and language where disciplinary, and intra- and intercultural biomedical *software program* (models of understanding) boundaries meet – a meta-science. I believe this might provide a core to the development of a global (communitarian cosmopolitan) biomedicine which in turn could guide a communitarian cosmopolitan bioethics. If the global political fact that is the hermeneutic circle, whereby without the whole the individual is less well understood as is the whole without the individual [13], is also the fact for biomedicine (as has been argued previously in this paper) then it follows that contemplating any aspect of any *software program* could be the starting point from which a meta-science might be revealed. My starting point began with the neuroscience of chronic pain. My intuitive thought was that neuroscientific exploration might explain the success of a clinical treatment modality for chronic pain. This resulted in a description of chronic pain in the form of an equation expressed by two parameters, activity and plasticity [14]; the underlying clinical objective being the creation of balance. This comports with Giordano’s understanding of the pain experience as occurring “through the activation of hierarchical networks that develop and may vary as a consequence of genotypic, phenotypic and environmental interactions throughout the lifespan of each individual” [15], as well as its subjective dimensions [16]. The next step was to understand the link between oral disease and a multitude of diseases that affect the various body systems. The explanation was provided by the psychoneuroimmunology community; there is but one system. It followed then that activity in any one of the nervous, immune or endocrine systems is activity in all three, the principles governing each system must be applicable to all, and homeostasis is the active balanced interaction between all three. This view brought the realisation that, theoretically at least, a single source of activity may find overt neural, immune and/or hormonal expression depending upon an individual’s biologic disposition. This is the reality in the varying bodily responses of individuals who encounter similar everyday external life stresses e.g. an academic exam.

Aligning with the suggestion that by stepping back from the molecular detail and considering the general behaviour of activity, plasticity and stability in biology, a role for macroscopic theory might be to reveal universal laws (a meta-science) in a living system governed by few degrees of variables [17, 18]. I present here a summary of the path through such macroscopic scientific theory based on psychoneuroimmunology with neuroplasticity at its centre:

Neuroscientifically the nervous system may be viewed as a series of ever changing activity thresholds the specifics of which are uniquely determined by a combination of each individual’s genotype and experience at any given moment in time. It is the modulation of activity by these thresholds that regulates the effect of any given activity at any given moment upon the multidimensional switching complex known as the genome. In turn, the effects of any given interaction modulates these activity thresholds. Activity is both excitatory and inhibitory, separately and simultaneously, and it is the combination of the level, character, timing and history of the activity that determines which characteristics of the nervous system are expressed. A simple illustration of this model in action: a nerve fibre depolarizes until the action potential threshold is reached whereupon an action potential is generated; action potentials accumulate up to the touch threshold whereupon touch is perceived; touch awareness increases with the applied pressure up to the pain threshold whereupon pain is suffered. The threshold levels might be thought of as switches and in this example they are effectively reversible, that is activity above the threshold - switch on, below the threshold – switch off.

Beyond the aforementioned artificial traditional Western biomedical boundaries revealed by psychoneuroimmunology, at the systems level where all activity is biomedical activity and all processes are biomedical processes, it follows that:

Integrative biomedicine might be described as a series of ever changing activity thresholds the specifics of which are uniquely determined by a combination of each individual’s genotype and experience at any given moment in time. It is the modulation of activity by these thresholds that regulates the effect of any given activity at any given moment upon the multidimensional switching complex known as the genome. In turn, the effects of any given interaction modulates these activity thresholds. Activity is both facilitatory and inhibitory, separately and simultaneously, and it is the combination of the level, character, timing and history of activity that determines which characteristics (signs and symptoms) are expressed.

Simplifying (mathematically to integrate is to simplify to a higher order); integrative biomedicine might be understood as the processing of the activity continuum that arises with conception and ceases with death, by a multidimensional series of switches, the nature of which vary with time and experience.

Simplifying further; integrative biomedicine = activity switch on / activity switch off.

Activity at one level is activity at all levels, sensitization in one system is sensitization in all systems, and either balance in toto or imbalance in toto.

Physical and psychosocial signs and symptoms are expressions of activity within an individual and are the messages (activity being the messenger) that reveal the state of that individual at any given moment. They may point to transient or more persistent activity, and indicate states of development, wellness and illness.

Although this description transcends many boundaries it remains, at the very least, linguistically and culturally constrained. Benedikter and Siepmann [13] stated that the multidimensional nature of globalization “is not just a world process, but also a process of awareness”. I posit that this is inclusive of a globalizing biomedicine. These authors also stated that “art experimentally outlines those basic tenets of what is to come … this is how art permanently generates an impact in the political context – whether it intends to do so or not.” They point to the possibility of a cosmopolitan art arising from the transdisciplinary interaction with intellectual and creative minds outside the domain of art. They question whether this art of globalization can be a catalyst for global awareness, and if so “which art and how exactly”? I answer in the affirmative and present a meta-science for biomedicine in poetry form:

*My essence is activitythe heart of me is balanceexpression through plasticityresultant are my talents *

(talents here can be defined as being all innate and acquired physical, psychosocial and, in some cases, spiritual characteristics of an individual at any given moment; activity might be measured by neural transmission in one biomedical *software program* and chi flow in another).

The hyper-complexity of the science is a potential hurdle to inter-, multi- and transdisciplinary deliberations. I suggest that from the higher order description (that is: integrative biomedicine = activity switch on / activity switch off) the following explanation might provide clarification:

Each biological organism may be viewed as an array of multidimensional switches through which all biological activity, whether intrinsically or extrinsically generated, is processed into the functional unit known as the individual. Each switch may be active or inactive and defined as being reversible (on/off), irreversible (switch on/stay on, switch off/stay off) or a combination of both at any given moment depending upon the level and nature of the ongoing activity and the history of all preceding activity. The expression of activity at any given scale (from molecular to in toto) will be determined by the combination of on and off switches which, theoretically, could be represented by binary code; this understanding could be named *digital biomedicine*.

### From hermeneutic circle to intelligible sphere – The discernment continuum

**Fig. 1:**
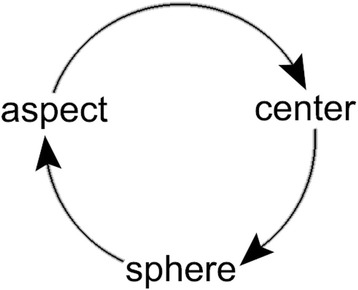
The discernment continuum

Benedikter and Siepmann [13] stated that globalization has seen the elision of borders and the ubiquitous everyday reality that we share the world with people from the most diverse cultures who have the most diverse world views. This “global systemic shift” in all six dimensions (economics, politics, culture, religion, technology and demographics) of modern differentiated, specialized societies brings with it inspiration and uncertainty in each. As globalization progresses global, national and local trends become more intermeshed resulting in a world order that is a “hyper-complex interplay of interrelated and overlapping elements with meanings that change fast as do spatio-temporal conditions” [13]. To address the urgent international issues that arise there must be an open, holistic understanding of the interactions between individuality and system, and we must think in process and not fixed structures. A methodology to achieving this may be provided by the Rawlsian perspective of “reflective equilibrium” as offered by Lanzilao et al. [2]. Inter-, multi- and trans-disciplinarity and their integration will be imperative. The challenges to such integrative discourse include the assumptions, orientations and limitations that each area brings [12] which are significantly expressed in the language of thought of each participant, whether it be disciplinary and/or mother tongue. I opine that to facilitate the participation of each and all as equal members in the process, a philosophical thought framework without boundaries will enhance the search for balance and inclusion (ethics) during the discourse, including when addressing the question central to all six dimensions, “what is the essence of the human being” [12]. I propose such a framework based on transforming the concept of the hermeneutic circle to an intelligible sphere with its center everywhere and circumference nowhere [19]. This then leads to the boundless conceptualization that is the discernment continuum (Fig. 1).

Rather than conceiving a discipline as being comprised of various systems, areas and levels, let it be thought of as a sphere^2^ of discernment. From any point of the sphere, known as an aspect of discernment, another sphere of discernment contained within the initial sphere of discernment may expand. An aspect of discernment from which a sphere of discernment expands will be known as a center of discernment. Expansion from any and every center of discernment will ultimately become the original sphere of discernment which may further expand; thus any beginning will expand into any and every other ad infinitum.

A biomedical example: choosing the aspect of discernment that is neuroscience as the center of discernment, it has expanded to incorporate the spheres of discernment immunology and endocrinology into the sphere of discernment integrative biomedicine. Here activity once thought neural, immune or hormonal has become biomedical; processes thought neuroscientific, immunological or endocrine are now biomedical. If we accept biomedical activity as the intersection point of the various intra- and intercultural biomedical *software programs*, and choose it as the center of discernment, then each *program* (sphere of discernment) will expand into the sphere of discernment that is global biomedicine. Within this framework the mind contemplating each sphere of discernment might recognise: (1) that it is an aspect of discernment of that sphere, that is a part of and not apart from the sphere; (2) the possible validity of other as yet not understood (to itself) biomedical understandings (spheres of discernment) and; (3) the boundaries within which it has evolved and in which it perceives. This may then enhance the ability for the self-evaluation, self-criticism, and self-revision (‘reflective equilibrium’) required for inclusive participation in discourse aimed at broadening perspective, as well as acknowledging and responding to the strengths and limitations of extant knowledge [12] necessary for the development of adequate global biomedicine and bioethics that enable both cosmopolitan and communitarian deliberation and application. These tools can then serve our “multiple-situated selves” within the varying communities and spheres of social space in which we live and participate [4].

### Communitarian cosmopolitan bioethics and biomedicine

Shook and Giordano [1] emphasised that “the objectivity of the new meta-ethics for neuroethics (now, as I assert, synonymous with bioethics) cannot exceed the degree of scientific objectivity involved.” They sought only intercultural principles to define universal norms inherent to cultures so that no one culture’s norms would be elevated to universalist status over humanity. I have sought similarly for the science so that it be liberated from the limitations of cultural imperialism, hence fortifying the robustness of the bioethics. To be valid, both the meta-ethics and meta-science must find seamless application from the local to the global, that is they must exhibit ‘communitarian cosmopolitanism’ as referred to by Dower [20]. ten Have defends the concept of ‘communitarian cosmopolitanism’ stating “the global sphere is not a domain in itself, separate from other specific domains. It includes them all, and at the same time manifests itself in each” … “The global is produced in the local” ..., “escapes the communities of its creation and is at the same time manifest in them” [4]. Lanzilao et al. note that this reflects “concentric circles (spheres) of moral concern (family, community, neighbourhood, nation, humanity) with the individual at the centre (of discernment)” [2]. I claim the aforementioned bioethics and biomedicine fulfil the required attributes and should be defined as communitarian cosmopolitan bioethics and communitarian cosmopolitan biomedicine respectively.

### Addressing the future

As we progress beyond the therapeutic applications of biomedicine and biotechnology to more radical modifications of human beings and the environment, we enter the third evolutionary stage, as described by Potter, in the development of ethics that is global bioethics, which deals with the relation of human beings with their environment, i.e. the “entire biological community” [4]. Shook and Giordano support such transformative bioethical adaption through evolutionary continuity between principled bioethics and medical ethics reconciling a principlism, “understood as the ethical prioritization of important moral ideals”, with pragmatism that permits a ‘reflective equilibrium’ approach when applying these ethical priorities to specific cases consistent with the scientific meta-ethics of bioethics [1]. Giordano, Hutchison and Benedikter assert that “the use of any ethical system in the absence of a core philosophy to provide moral grounding will be necessarily hollow and subject to bastardization” [21]. Through classical philosophical reflection, Pellegrino developed a theory of medicine based on what medicine is in reality, initially asking ‘what is the end, the telos, of medicine?’;“in the classical sense of the end as that from which an activity exists, and that which when attained constitutes a good.” He constructed a philosophy of medicine that defines medicine’s primary end as “healing and helping”, and noted the ethical obligation across centuries and cultures all focus on the ethical primacy of the welfare of the sick person [22, 23]. Comporting with and advancing this approach, Hauskeller advocates for the revival of the Aristotelian concept of telos to deal with the present day ethics of the modification of living beings, since it considers internal ends and therefore an awareness of an essential part of what it means to be a living being, including humans [24]. Reflection upon the classical approach evolving medical ethics into global bioethics will require the ‘discovery’ [21] of a telos (specific ‘internal’ end) of humankind, in which to ground an ‘internal’ morality of humankind. I begin the path to ‘discovery’ by reflecting upon medicine’s primary end, followed by observations of a real-world situation and then redefine “healing and helping” [22] in synonymous terms.

Solbakk engaged in a Socratic exploration of the question, “What is it to do good medical ethics?” and directly related both a ‘functional’ and an ‘hedonic’ answer to the alleviation of suffering [25]. In their exploration of the biopsychosocial model of clinical care, Borrell-Carrió, Suchman and Epstein calibrate the skill of a physician (to do good) based on their ability to “relieve the patient’s suffering” [11].

Chambers contends that narrative theory should be thought of as being as vital to bioethics as moral theory. He states that recognizing the importance that cases have for the way bioethics is done is “essential in order to understand the field as a form of applied philosophy” [26]. I offer the following real-world narrative:

In the public dental clinic in which I work, we regularly attend to patients in extreme physical and/or psychosocial pain. Provision of a biopsychosocially oriented clinical approach [11] delivers significant relief to many. Successfully helping a patient evokes an involuntary positive sense of worth in involved clinical and non-clinical staff, both proximal and distant to the clinical interaction. Conversely, when we are unable to help, a sense of despondency descends. These emotions arise spontaneously and when questioned regarding their feelings the staff directly attribute this to our ability, or not, to alleviate the patient’s suffering. Such emotions arise whether or not treatment has yet been provided, and even when successfully helping or not is only is only a theoretical thought, that is the emotions are independent of whether the patient is real or ‘virtual’.

At this moment on our reflective evolutionary path, I contend that the telos of medicine, “healing and helping”, can equally be defined as the *alleviation of suffering*.

I observe that the above real-world narrative offers further insight. The ability or not to provide, actual or prospective, real or virtual *alleviation of suffering* evokes similar emotional responses equally in the healing professional, administrative staff, non-professional staff, individual patient, their accompanying persons, unrelated patients and, outside the clinical precinct, in those to whom stories of such outcomes are told.

This raises the question, is the *alleviation of suffering* an intrinsic end of humankind, i.e. a good of and for human beings? Reflecting across the narratives of human history, real or imagined, superficial or deep, transcending time, culture, and geography the *logos* appears to be the same, the *alleviation of suffering*. At one extreme the narrative may focus narrowly on the individual and their personal physical and/or psychosocial suffering, at the other it may encompass all aspects of the concentric spheres of moral concern (family, community, neighbourhood, nation, humanity), past, present and future, in the physical and metaphysical realms, with the individual as the centre of discernment.

I contend that the telos (the primary end [good]) of humankind is the *alleviation of suffering*.

This along with the previously defined meta-science, which I argue provides a realist account of biomedicine – what biomedicine *is* rather than what happens in biomedicine – that “does not change with changing circumstances, in different locations, or with different people”(or living beings) [21], provide both core philosophy and science which, in conjunction with the principled, communitarian cosmopolitan neuroethics proposed by Shook and Giordano [1], and Lanzilao et al. [2], evolve into global (biomedical) bioethics. Applying a Rawlsian perspective to this offers a methodology to develop inclusive inter-, multi-, and trans-disciplinary inter-cultural understandings and strategies to address the urgent international governance issues around current and future globalizing bio-medical/technology research and applications.

## Conclusion

When discussing the role of bioethics, Solbakk [25] emphasises the importance of distinguishing between genuine inclusive dialogue with each participant on equal terms, and manipulative rhetoric aimed at coercing consensus by declaring ‘the good’ based on the most powerful, or most vocal, or most Weternised, etc., socio-cultural *external* construct. This is echoed by Giordano, Hutchison and Benedikter [21] who exhort us to look beyond the “market-place” with its dehumanising, socially prescriptive, economically based, proclamation of human ‘good’ as being “competition” [27]. It is reflected by Engel [10, 28], Borrell-Carrio, Suchman and Epstein [11], and Stein and Giordano [29] who caution against materialistic, reductionistic and technically oriented biomedical models that neglect the human dimension. By aligning with and building upon a new meta-ethics for neuroethics [1, 2] through the incorporation of a biomedical meta-science and classically derived telos of human-kind, I offer a global biomedical bioethics that is morally and scientifically grounded in what humankind *is* rather than does so that as we progress deeper into the Biomedical (21st) Century [30], we as individuals, communities and a species can reflect upon and develop a deeper realisation and appreciation of who we are and what we are and then, by modelling possibilities through a variety of perspectives, we might critically choose what we become. To inform and facilitate the application of this meta-ethics and the four guidelines to the entire biomedical-science/technology sphere I have offered three provisions. First: an integrative western based biomedical model that provides an integrative systems scientific foundation which transcends traditional western biomedical boundaries. Second: a meta-science where the many and varied biomedical software programs might intersect in common language enabling each and all to participate as equal members. Third: a thought framework to facilitate thorough and balanced reflection, one that encourages an ever broadening awareness as the globalization process progresses. The first two provisions lead to inter-cultural scientific principles as the foundation for a principled and cosmopolitan biomedicine which then may inform a principled and cosmopolitan bioethics synonymous to the aforementioned neuroethics. In conjunction with the third provision, inclusive inter-, multi-, and transdisciplinary inter-cultural discourse may be facilitated and enhanced to deal with the current and future issues facing humanity from the global advances in biomedical and biotechnology research and their uses.

## Methods

A philosophical methodology incorporating novel and classical philosophical reflection was used.

## Footnotes

1 For a review of the psychoneuroimmunoloy science I suggest the 2007 Named Series of articles: “Twenty Years of Brain, Behavior, and Immunity” in the journal of the same name

2 sphere may be synonymous with dimension
